# Rotavirus and Severe Childhood Diarrhea

**DOI:** 10.3201/eid1202.050006

**Published:** 2006-02

**Authors:** Umesh D. Parashar, Christopher J. Gibson, Joseph S. Bresee, Roger I. Glass

**Affiliations:** * Centers for Disease Control and Prevention, Atlanta, Georgia, USA

**Keywords:** Diarrhea, rotavirus, mortality, morbidity, hospitalizations, disease burden, dispatch

## Abstract

Studies published between 1986 and 1999 indicated that rotavirus causes ≈22% (range 17%–28%) of childhood diarrhea hospitalizations. From 2000 to 2004, this proportion increased to 39% (range 29%–45%). Application of this proportion to the recent World Health Organization estimates of diarrhea-related childhood deaths gave an estimated 611,000 (range 454,000–705,000) rotavirus-related deaths.

Rotavirus is the leading cause of diarrhea hospitalization among children worldwide (1). In 2003, we published an estimate of rotavirus-related deaths worldwide based on a review of the literature published from 1986 through 1999 on deaths caused by diarrhea and rotavirus hospitalizations in children (*2*). This review indicated that rotavirus accounted for ≈22% of hospitalizations for childhood diarrhea. By applying this fraction to an estimate of 2.1 million annual deaths from diarrhea, we calculated that rotavirus causes 440,000 annual deaths in children <5 years of age worldwide. This estimate was ≈50% of the 1985 estimate of 873,000 rotavirus deaths per year (*3*), and the decrease in estimated rotavirus-related deaths paralleled the decrease in deaths from diarrhea of all causes from an estimated 4.6 million deaths in 1982 to 1.6–2.5 million deaths in 2000 (*4**–**6*).

Recent studies suggest that as global deaths from childhood diarrhea decreased during the past 2 decades, the proportion of diarrhea hospitalizations attributable to rotavirus may have increased. For example, prospective, sentinel hospital–based surveillance of rotavirus disease in 9 Asian countries demonstrated a median rotavirus detection of 45% among children hospitalized with diarrhea (*7*), a figure that was considerably greater than the detection rates in previous studies from the same countries. Similarly, a more extensive study of 5,768 children hospitalized from 1998 through 2000 in 6 centers in Vietnam identified rotavirus in 56% of patients (*8*), a proportion that was more than twice the 21% detection rate reported among children hospitalized with diarrhea in a hospital in Hanoi, Vietnam, from 1981 to 1984 (*9*).

To systematically evaluate whether these recent reports are isolated observations or reflect a changing trend in the etiology of childhood diarrhea hospitalizations, we reviewed studies of rotavirus detection among children hospitalized with diarrhea published from 2000 through 2004 and compared the data with those of the previous review of studies published from 1986 through 1999.

## The Study

Similar to the approach used in our previous review, we performed a computer search of the scientific literature (in English and other languages) published from January 2000 through June 2004 using the words rotavirus and the truncated stem rota-. We restricted the analysis to studies that met the following criteria: 1) were initiated after 1993; 2) were conducted for at least 1 full calendar year; and 3) examined rotavirus among at least 100 children <5 years of age hospitalized with diarrhea.

For each study, we determined the proportion of cases positive for rotavirus among children hospitalized with diarrhea. We plotted this proportion against the per capita gross national product (GNP) for the country in which the study was conducted. We then classified countries by per capita GNP into World Bank income groups (low, <US $756; low-middle, US $756–$2,995; high-middle, US $2,996–$9,265; and high, >US $9,265) (*10*), and calculated the median (interquartile range [IQR]) proportion of diarrhea hospitalizations attributable to rotavirus for each income group.

We next calculated an overall median detection rate by taking a weighted average of the median detection rates for each of the income groups. The weights assigned to each income group corresponded to the proportion of deaths from childhood diarrhea among countries in that income group as determined on the basis of our previous analysis (*2*): 85% in low-income countries, 13% in low-middle–income countries, 2% in high-middle–income countries, and <1% in high-income countries. To estimate deaths from rotavirus disease among children, we multiplied the overall median detection rate of rotavirus among children hospitalized with diarrhea by a recent World Health Organization estimate of deaths from diarrhea among children worldwide (*5*).

**Figure 1:**
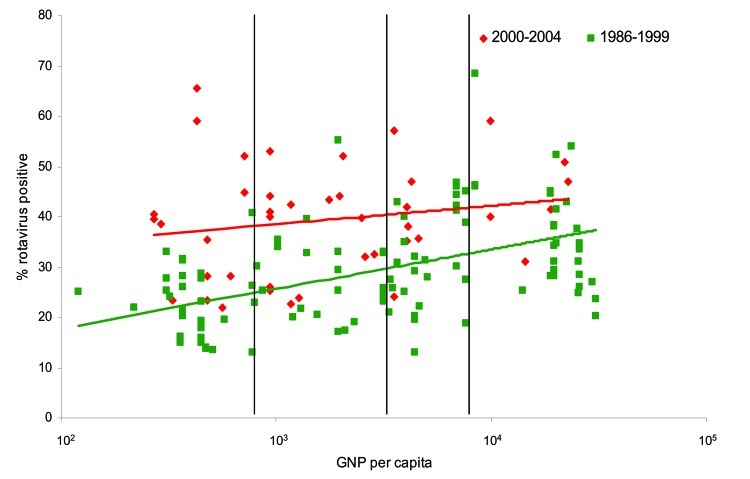
Percentage of severe diarrhea cases attributable to rotavirus for countries in different World Bank income groups, by per capita gross national product (GNP), for studies published in 1986–1999 and 2000–2004. GNP is in US dollars. Upper line, trend for 2000–2004; lower line, trend for 1986–1999.

We abstracted information from 41 studies that met all the inclusion criteria (Table A1). Unlike the previous review of studies conducted for the period 1986–1999, in which the proportion of diarrhea-related hospitalizations attributable to rotavirus showed a distinct increasing trend with increasing income level, we found that the median detection rates increased only slightly with increasing income level (Figure 1). The median detection rate for rotavirus among children hospitalized with diarrhea was 39% in studies conducted in low-income countries, 40% for low-middle–income countries, 38% for high-middle–income countries, and 44% for high-income countries, for an overall weighted median estimate of 39% (Table).

**Table:** Percentage of diarrhea hospitalizations attributable to rotavirus for countries in different World Bank income groups, 1986–1999 and 2000–2004

Income group	Median % (interquartile range) of diarrhea-associated hospitalizations due to rotavirus	
	1986–1999	2000–2004
Low	20 (16–27)	39 (28–45)
Low middle	25 (20–33)	40 (32–43)
High middle	31 (25–42)	38 (35–45)
High	34 (28–38)	44 (40–50)
Total*	21 (17–28)	39 (29–45)

*The overall median was calculated by taking a weighted average of the median rotavirus detection rate for each income group. The weights applied to each group corresponded to that group's proportion of global diarrheal deaths: 85% for low-income countries, 13% for low-middle–income countries, 2% for high-middle–income countries, and <1% for high-income countries.

**Figure 2:**
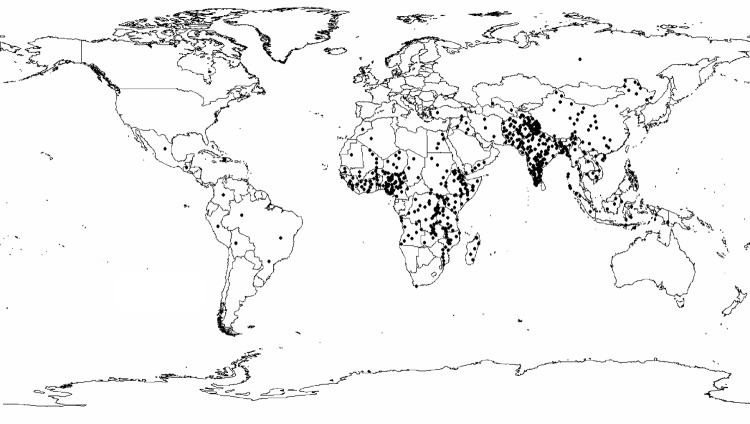
Estimated global distribution of rotavirus-related deaths. Each dot represents 1,000 rotavirus-related deaths.

If we multiply the greater median rotavirus detection rate of 39% (IQR 29%–45%) from this analysis by 1,566,000 recently estimated childhood diarrhea deaths (*5*), we find that rotavirus causes ≈611,000 childhood deaths (IQR 454,000–705,000). More than 80% of all rotavirus-related deaths were estimated to occur in low-income countries of south Asia and sub-Saharan Africa (Figure 2).

## Conclusions

Compared with results from studies published from 1986 to 1999, the proportion of diarrhea hospitalizations attributable to rotavirus appears to have increased between 2000 and 2004. This phenomenon likely reflects a relatively slower rate of decrease in hospitalizations for rotavirus compared with other causes of severe childhood diarrhea. This finding could be accounted for by several factors. First, interventions to improve hygiene and sanitation are likely to have a greater impact on diarrhea caused by bacterial and parasitic agents, which are transmitted primarily through contaminated food or water, unlike rotavirus, which is often spread from person-to-person. This hypothesis is supported by data from the United States (*11*) and Mexico (*12*), which showed that as diarrhea-related childhood deaths decreased dramatically in both countries; the decrease was greatest during the summer months when diarrheal diseases caused by bacteria are more prevalent. In both countries, diarrhea-related deaths in recent years have exhibited peaks only in the winter when rotavirus infections are common. Second, oral hydration therapy to replace loss of body fluids, which many regard as a major factor responsible for the decrease in diarrhea deaths (*13*), is often more difficult to successfully administer in children with severe vomiting (*14*), a common manifestation of rotavirus disease. Third, unlike antimicrobial therapies that are effective against some bacterial and parasitic agents, no specific treatment for rotavirus infection is available.

We have derived preliminary updated estimates of rotavirus-related childhood deaths on the basis of the findings of our review. Because we wanted to assess the most recent trends in rotavirus incidence, we examined a relatively limited number of studies published in the last 5 years, particularly from upper-middle– and high-income countries. However, these 2 income groups account for only a small fraction (<5%) of all deaths from rotavirus disease, and the 28 studies available from low- and low-middle–income countries allowed for a reasonably robust analysis. Nevertheless, our findings should be updated as new data on rotavirus hospitalizations and updated estimates of childhood diarrhea deaths become available. In 2002, the World Health Organization published a generic protocol for hospital-based surveillance of rotavirus (*15*), and studies using this protocol are currently being conducted or planned in >30 countries in Asia, Africa, the Middle East, and Latin America. Data from these and other studies, particularly from countries such as India and China, which account for a large fraction of global rotavirus deaths, should be used to update our estimate of rotavirus-related deaths and further refine it to develop country-specific figures. These data, together with information on effects and costs of rotavirus disease, will allow policymakers to assess the magnitude of the problem of rotavirus and the value of new vaccines that may soon be available.

## Appendix

**Table A1:** Characteristics of studies on worldwide diarrheal deaths and hospitalizations, 2000–2004

Income group and country	Year of publication	Study period	No. diarrhea hospitalizations	Rotavirus detection rate (%)	Reference
Low					
Cameroon	2003	1999–2000	890	21.9	16
Côte d'Ivoire	2003	1997–1999	479	28.3	17
Ghana	2003	1997–2002	2,205	40.5	18
Ghana	2003	1998–2000	1,717	39.4	19
India	2004	2001	401	35.5	20
India	2001	1993–1996	628	28.3	21
India	2003	1999–2000	202	23.3	22
Indonesia	2004	2001–2002	577	52.0	23
Indonesia	2002	1997–1999	339	44.8	24
Nigeria	2003	1998–1999	417	38.6	17
Vietnam	2004	2001–2002	1,570	59.0	23
Vietnam	2003	1999–2000	1,355	65.6	25
Zambia	2003	1997–1999	1,635	23.3	17
Low-middle					
Bosnia-Herzegovina	2003	1999–2000	201	23.9	26
Brazil	2001	1998–1999	190	32.6	27
China	2004	2001–2002	2,079	44.0	23
China	2000	1996–1999	1,130	26.1	28
China	2002	1998–2000	3,177	41.0	29
China	2003	1998–2001	1,211	52.9	30
China	2004	1999–2001	1,230	40.1	31
Jordan	2002	1997–2000	840	43.4	32
Paraguay	2003	1998–2000	410	22.7	33
Paraguay	2002	1999–2000	141	42.5	34
Peru	2001	1995–1997	386	52.0	35
South Africa	2003	1997–1999	1,525	32.0	17
Thailand	2004	2001–2002	992	44.0	23
Turkey	2003	2000–2001	920	39.8	36
High-middle					
Argentina	2001	1996–1998	1,312	42.0	37
Argentina	2001	1997–1998	133	35.3	38
Chile	2001	1997–1999	276	47.0	39
Malaysia	2004	2001–2002	1,374	57.0	23
Malaysia	2003	1996–1999	1,362	24.0	40
Poland	2000	1997	773	35.6	41
Venezuela	2001	1997–1999	946	38.0	39
High					
France	2002	1997–2000	706	50.9	42
Germany	2003	2001–2002	217	47.0	43
Italy	2002	2001	330	41.4	44
South Korea	2000	1998–1999	243	40.0	45
South Korea	2003	1998–2000	348	59.0	46
Spain	2004	1998–2002	3,760	31.0	47
